# Racial/ethnic disparities in the distribution and effect of type and number of high-risk criteria on mortality in prostate cancer patients treated with radiotherapy

**DOI:** 10.1080/2090598X.2022.2148867

**Published:** 2022-11-21

**Authors:** Francesco Chierigo, Rocco Simone Flammia, Gabriele Sorce, Benedikt Hoeh, Lukas Hohenhorst, Andrea Panunzio, Zhe Tian, Fred Saad, Markus Graefen, Michele Gallucci, Alberto Briganti, Francesco Montorsi, Felix K.H. Chun, Shahrokh F. Shariat, Alessandro Antonelli, Giovanni Guano, Guglielmo Mantica, Marco Borghesi, Nazareno Suardi, Carlo Terrone, Pierre I. Karakiewicz

**Affiliations:** a Department of Urology, IRCCS Ospedale Policlinico San Martino, Genova, Italy; b Cancer Prognostics and Health Outcomes Unit, Division of Urology, University of Montréal Health Center, Montréal, Québec, Canada; c Department of Surgical and Diagnostic Integrated Sciences (DISC), University of Genova, Genova, Italy; d Department of Maternal-Child and Urological Sciences, Sapienza Rome University, Policlinico Umberto I Hospital, Rome, Italy; e Division of Experimental Oncology/Unit of Urology, URI, Urological Research Institute, IRCCS San Raffaele Scientific Institute, Milan, Italy; f Department of Urology, University Hospital Frankfurt, Frankfurt am Main, Germany; g Martini-Klinik Prostate Cancer Center, University Hospital Hamburg-Eppendorf, Hamburg, Germany; h Department of Urology, University of Verona, Azienda Ospedaliera Universitaria Integrata di Verona; i Department of Urology, Comprehensive Cancer Center, Medical University of Vienna, Vienna, Austria; j Departments of Urology, Weill Cornell Medical College, New York, NY, USA; k Department of Urology, University of Texas Southwestern, Dallas, Texas, USA; l Department of Urology, Second Faculty of Medicine, Charles University, Prague, Czech Republic; m Institute for Urology and Reproductive Health, I.M. Sechenov First Moscow State Medical University, Moscow, Russia; n Division of Urology, Department of Special Surgery, Jordan University Hospital, The University of Jordan, Amman, Jordan

**Keywords:** radiotherapy, race/ethnicity, CSM, D’Amico high-risk criteria, SEER

## Abstract

**Objective:**

To assess differences in the distribution of type and number of D’Amico high-risk criteria (DHRCs) according to race/ethnicity (R/E) and their effect on cancer-specific mortality (CSM) in prostate cancer (PCa) patients treated with external beam radiotherapy (RT).

**Methods:**

In the SEER database (2004–2016), we identified 31,002 PCa patients treated with RT with at least one DHRCs, namely PSA >20 ng/dL, biopsy Gleason Grade Group 4–5, and clinical T stage ≥T2c. Competing risks regression (CRR) model tested the association between DHRCs and 5-year CSM in all R/E subgroups.

**Results:**

Of 31,002 patients, 20,894 (67%) were Caucasian, 5256 (17%) were African American, 2868 (9.3%) were Hispanic-Latino, and 1984 (6.4%) were Asian. The distributions of individual DHRCs and combinations of two DHRCs differed according to R/E, but not for the combination of three DHRCs. The effect related to the presence of a single DHRC, and combinations of two or three DHRCs on absolute CSM rates was lowest in Asians (1.2–6.8%), followed by in African Americans (2.3–12.2%) and Caucasians (2.3–12.1%), and highest in Hispanic/Latinos (1.7–13.8%). However, the opposite effect was observed in CRR, where hazard ratios were highest in Asians vs. other R/Es: Asians 1.00–2.59 vs. others 0.5–1.83 for one DHRC, Asians 3.4–4.75 vs. others 0.66–3.66 for two DHRCs, and Asians 7.22 vs. others 3.03–4.99 for all three DHRCs.

**Conclusions:**

R/E affects the proportions of DHRCs. Moreover, within the four examined R/E groups, the effect of DHRCs on absolute and relative CSM metrics also differed. Therefore, R/E-specific considerations may be warranted in high-risk PCa patients treated with RT.

## Introduction

The risk stratification system, proposed by D’Amico et al. in 1998, classifies patients into low-, intermediate-, and high-risk (HR) groups based on clinical tumor stage (cT), prostate-specific antigen (PSA) level, and biopsy Grade Group (GG) at diagnosis [1]. It is widely used for clinical decision-making in newly diagnosed localized prostate cancer (PCa) patients.

According to the D’Amico classification, 30% of newly diagnosed clinically localized PCas are defined as HR (clinical stage ≥T2c, and/or PSA >20 ng/mL, and/or biopsy GG 4–5) [2,3]. An important degree of heterogeneity may exist within HR PCa patients based on number and type of HR criteria that they harbor. Specifically, a stepwise increase in risk may exist based on the type and number of HR criteria.

Previous evidence indicates differences in PCa phenotype according to race/ethnicity (R/E). Specifically, in a study by Mahal et al., Black men were twice as likely to die of PCa compared with non-Black men [4]. Moreover, Steele et al. reported racial/ethnic disparities in receipt of definite treatment and cancer-specific survival [5]. Finally, the research group lead by Pierre Karakiewicz focused on racial/ethnic differences in survival according to stage and treatment received [6–9].

We hypothesized that such racial/ethnic differences might also apply to the distribution of type (clinical T stage T2c–T3, PSA >20 ng/mL, and biopsy Gleason GG (GGG) 4–5) and number (one, vs. combinations of two, vs. all three) of D’Amico high-risk criteria (DHRC). For example, it is possible that a larger proportion of African American patients may harbor three concomitant DHRCs than Caucasians. Moreover, we also postulated that the effect of individual and/or combinations of two or three DHRCs may affect cancer-specific mortality (CSM) differently, according to R/E. For example, it is possible that concomitant presence of two or three DHRCs may predispose African Americans to higher CSM than Caucasians, after multivariable adjustment for baseline PCa characteristics. We tested the above hypotheses in localized HR PCa patients treated with external beam radiotherapy (RT) within the Surveillance, Epidemiology, and End Results (SEER) database (2004–2016).

## Material and methods

### Study population

The SEER database samples 26% of the United States and approximates the United States in terms of demographic composition, as well as cancer incidence [10]. Within the SEER database 2004−2016, we focused on four R/E groups: Caucasians, African Americans, Hispanic/Latinos, and Asians, aged ≥18 years old, with histologically confirmed non-metastatic adenocarcinoma of the prostate, diagnosed at biopsy (International Classification of Disease for Oncology [ICD-O-3] code 8140 site code C61.9), which fulfilled DHRC (defined as biopsy GGG 4–5, and/or PSA >20 ng/mL, and/or clinical stage ≥T2c), treated with RT. Patients with unknown clinical stage or clinical T4 stage, unknown biopsy GGG, unknown PSA or PSA >50 ng/mL, as well as autopsy/death-certificate-only cases were excluded.

CSM was defined as deaths attributable to PCa. Conversely, other cause mortality (OCM) was defined as deaths attributable to other causes than PCa. The exact cause of death was obtained from death certificates, which are coded by the state health department or state vital records.

Follow-up was defined as time from diagnosis to CSM, OCM, loss to follow-up, or end of study. Censoring occurred at end of the available observation unless the event of interest (CSM or OCM) occurred.

### Statistical analyses

Descriptive statistics included proportions and frequencies for categorical variables. Means and interquartile ranges (IQRs) were reported for continuously coded variables. The Chi-square tested the statistical significance in proportions’ differences. The *t*-test and Kruskal–Wallis test examined the statistical significance of means and distributions’ differences. The endpoint of interest was CSM. We relied on cumulative incidence plots and competing risks regression models, where CSM rates were adjusted for OCM, to test the association between 5-year CSM rates and the type and number of DHRCs. All analyses were conducted for each individual R/E. For all statistical analyses, tests were two sided with a level of significance set at *p* < .05, and R software environment for statistical computing and graphics (version 3.4.3) was used [11].

## Results

### Study population

We identified 31,002 HR PCa patients treated with RT (Table 1). Of these, 20,894 (67%) were Caucasian, 5256 (17%) were African American, 2868 (9.3%) were Hispanic, and 1984 (6.4%) were Asian.

**Table 1.** Descriptive characteristics of 31,002 non-metastatic D’Amico high-risk prostate cancer patients treated with external beam radiotherapy, stratified according to race/ethnicity, within the Surveillance, Epidemiology and End Results (2004–2016) database.

	Race/Ethnicity				
Characteristic	Caucasian, N = 20,894 (67%)^1^	African-American, N = 5256 (17%)^1^	Hispanic/Latino, N = 2868 (9.3%)^1^	Asian, N = 1984 (6.4%)^1^	*p*-Value^2^
**Age, median (IQR)**	72 (66, 77)	67 (61, 72)	70 (65, 76)	73 (67, 77)	<0.001
**PSA, median (IQR)**	10 (6, 20)	14 (7, 25)	13 (7, 24)	11 (7, 22)	<0.001
**Biopsy GGG, n (%)**					<0.001
1	1,641 (7.9%)	538 (10%)	294 (10%)	128 (6.5%)	
2	2,508 (12%)	886 (17%)	385 (13%)	214 (11%)	
3	1,969 (9.4%)	552 (11%)	289 (10%)	172 (8.7%)	
4	8,641 (41%)	2,077 (40%)	1,160 (40%)	909 (46%)	
5	6,135 (29%)	1,203 (23%)	740 (26%)	561 (28%)	
**Clinical T stage, n (%)**					<0.001
T1c-T2a	9,751 (47%)	3,155 (60%)	1,381 (48%)	1,021 (51%)	
T2b	4,595 (22%)	774 (15%)	750 (26%)	469 (24%)	
T2c-T3	6,548 (31%)	1,327 (25%)	737 (26%)	494 (25%)	
**Type and number of HR criteria, n (%)**					
cT2c-3	3,090 (15%)	670 (13%)	351 (12%)	204 (10%)	<0.001
PSA >20	2,500 (12%)	1,120 (21%)	540 (19%)	270 (14%)	<0.001
GGG 4–5	10,226 (49%)	2,245 (43%)	1,303 (45%)	1,003 (51%)	<0.001
PSA+cT	528 (2.5%)	186 (3.5%)	77 (2.7%)	40 (2.0%)	<0.001
PSA+GGG	1,620 (7.8%)	564 (11%)	288 (10%)	217 (11%)	<0.001
cT+GGG	2,257 (11%)	303 (5.8%)	211 (7.4%)	180 (9.1%)	<0.001
PSA+cT+GGG	673 (3.2%)	168 (3.2%)	98 (3.4%)	70 (3.5%)	0.8

^1^Median (IQR); n (%).

^2^Kruskal–Wallis rank sum test; Pearson’s chi-squared test.

Age, PSA, clinical T stage, and biopsy GGG distributions exhibited statistically significant differences among the four examined R/Es (Table 1). Asians were oldest (median age 73 years), exhibited the lowest rate of T2c-T3 (25%), the second lowest median PSA (11, IQR 7–22), the highest GGG 4 (46%), and second highest GGG 5 rates (28%).

The distribution of type and number of DHRCs varied with R/E. Statistically significant differences were recorded with respect to the presence of a single DHRC or combinations of two concomitant DHRCs (Table 1 – Supplementary Figure S1). Regarding individuals with a single DHRC, the rate of GGG 4–5 was highest in Asians (51%). Conversely, PSA >20 ng/mL was highest in African Americans (21%) and second lowest in Asians (14%). Moreover, the rate of clinical stage T2c-T3 was highest in Caucasians (15%) and lowest in Asians (10%). Regarding individuals with two DHRCs, the concomitant presence of PSA >20 ng/mL and GGG 4–5 was lowest in Caucasians (7.8%), but strikingly similar in African Americans (11%), Hispanic/Latinos (10%), and Asians (11%). Conversely, the concomitant presence of T2c-T3 and GGG 4–5 was lowest in African Americans (5.8%), intermediate in Hispanic/Latinos (7.4%), and highest in Asians (9.1%) and Caucasians (11%). No clinically meaningful differences were recorded for the concomitant presence of PSA >20 ng/mL and T2c-T3 (2.0–3.5%). Finally, no statistically significant or clinically meaningful differences according to R/E were recorded for the concomitant presence of three DHRCs.

### Absolute mortality rates according to race/ethnicity

Cumulative incidence plot-derived 5-year CSM rates exhibited important differences in absolute CSM values, according to R/E. These differences also applied to specific CSM values, according to presence of one, combinations of two, and all three DHRCs (Table 2). Specifically, lowest 5-year CSM rates were recorded in presence of one DHRC. Within R/Es, the lowest 5-year CSM for the presence of one DHRC was recorded in Asians (1.2–2.5%), followed by Hispanic/Latino (1.6–4.7%), African Americans (2.3–4.4%), and Caucasians (2.3–4.9%), in that order. The concomitant presence of two DHRCs yielded intermediate CSM values, which were invariably situated between those recorded for the presence of one and the presence of three DHRCs. Within R/Es, the lowest 5-year CSM for the concomitant presence of two DHRCs was recorded in Asians (2.8–4.4%), followed by Caucasians (4.3–9.3%), African Americans (5.2–10.6%), and Hispanic/Latinos (1.6–12.2%), in that order. However, for the concomitant presence of PSA >20 ng/mL and cT2c-T3, CSM rates were lowest in Hispanic/Latinos (1.6% vs. 2.8–5.2%). Finally, the presence of all three DHRCs yielded the highest CSM rates. Within R/Es, the CSM rates for the concomitant presence of all three DHRCs were also lowest in Asians (6.8%), followed by African Americans (12.2%), Caucasians (12.1%), and Hispanic/Latinos (13.8%), in that order.

**Table 2.** Tabulation of cumulative incidence plot-derived 5-year cancer-specific mortality (CSM) rates according to type and number of high-risk criteria, in D’Amico high-risk prostate cancer (PCa) patients treated with external beam radiotherapy. Rates are stratified according to race-ethnicity.

	Cumulative incidence – CSM			
	Caucasian	African-American	Hispanic-Latino	Asian
cT2c-3	2.3%	2.3%	1.7%	1.2%
PSA >20	2.6%	2.2%	1.6%	1.9%
GGG 4–5	4.9%	4.4%	4.7%	2.5%
PSA+cT	4.3%	5.2%	1.6%	2.8%
PSA+GGG	9.3%	7.9%	7.2%	2.4%
cT+GGG	8.6%	10.6%	12.2%	4.4%
cT+PSA+GGG	12.1%	12.2%	13.8%	6.8%

Regarding OCM rates, we also recorded R/E-specific differences. Specifically, OCM rates were 12.8% for Caucasians, 13.3% for African Americans, 9.7% for Hispanic-Latinos, and 8.6% for Asians.

### Competing risks regression-derived mortality rates according to race/ethnicities

**Figure 1.**
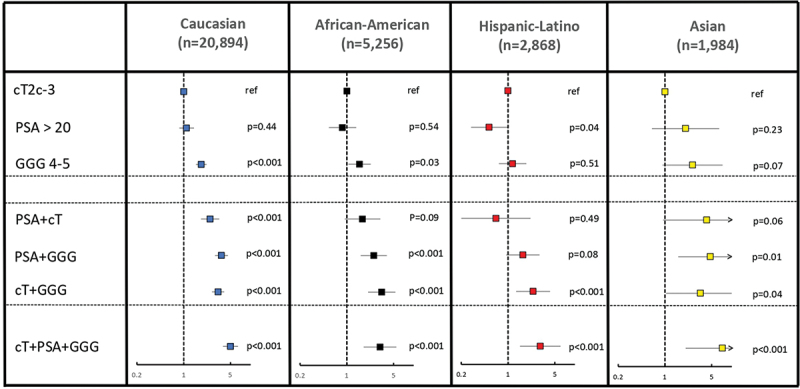
Forest plots depicting hazard ratios, their respective 95% confidence intervals, and *p*-values of the competing risks regression models testing the association between cancer-specific mortality (CSM), after adjustment for other cause mortality (OCM), and type and number of high-risk criteria in D’Amico high-risk prostate cancer (PCa) patients treated with external beam radiotherapy. All analyses were stratified according to race/ethnicity.

Competing risks regression-derived hazard ratios predicting CSM according to type and number of DHRCs exhibited important differences according to R/E (Table 3, Figure 1). These differences also applied to specific CSM values, according to presence of one, combinations of two, and all three DHRCs. Specifically, lowest hazard ratios were recorded in the presence of one DHRC. Within R/Es, the highest hazard ratios for the presence of one DHRC were recorded in Asians, followed by Caucasians, African Americans, and Hispanic/Latinos, in that order. Specifically, compared to cT2c-3, PSA >20 ng/mL yielded to hazard ratios (95% confidence interval (CI)) of 2.03 (0.64–6.48) for Asians, 1.10 (0.86–1.42) for Caucasians, 0.86 (0.54–1.38) for African Americans, and 0.52 (0.28–0.99) for Hispanic-Latinos. Similarly, the presence of biopsy GGG 4–5 yielded to hazard ratios of 2.59 (0.92–7.29) for Asians, 1.83 (1.52–2.2) for Caucasians, 1.55 (1.05–2.28) for African Americans, and 1.17 (0.73–1.88) for Hispanic-Latinos. The concomitant presence of two DHRCs yielded intermediate hazard ratios, which were invariably situated between those recorded for the presence of one and the presence of all three DHRCs. Within R/Es, the highest hazard ratios were recorded in Asians, followed by Caucasians, African Americans, and Hispanic/Latinos, in that order (Table 3, Figure 1). Finally, the concomitant presence of all three DHRCs yielded the highest hazard ratios. Within R/Es, hazard ratio for the concomitant presence of all three DHRCs was also highest in Asians (7.22, 95% CI: 2.04–25.51), followed by Caucasians (4.99, 95% CI: 3.85–6.47), African Americans (3.14, 95% CI: 1.78–5.52), and Hispanic/Latino (3.03, 95% CI: 1.51–6.09), in that order. All of the above hazard ratios were adjusted according to OCM.

**Table 3.** Competing risks regression analysis to test the association between cancer-specific mortality (CSM), after adjustment for other cause mortality (OCM), and type and number of high-risk criteria, in D’Amico high-risk prostate cancer (PCa) patients treated with external beam radiotherapy. Rates are stratified according to race-ethnicity.

	Competing-risks regression							
	Caucasian		African-American		Hispanic-Latino		Asian	
	HR (95% CI)	*p*-Value	HR (95% CI)	*p*-Value	HR (95% CI)	*p*-Value	HR (95% CI)	*p*-Value
cT2c-3	ref		ref		ref		ref	
PSA >20	1.10 (0.86–1.42)	0.44	0.86 (0.54–1.38)	0.54	0.52 (0.28–0.99)	0.04	2.03 (0.64–6.48)	0.23
GGG 4–5	1.83 (1.52–2.2)	<0.001	1.55 (1.05–2.28)	0.03	1.17 (0.73–1.88)	0.51	2.59 (0.92–7.29)	0.07
PSA+cT	2.47 (1.81–3.39)	<0.001	1.71 (0.93–3.17)	0.09	0.66 (0.2–2.17)	0.49	4.20 (0.94–18.64)	0.06
PSA+GGG	3.66 (2.93–4.57)	<0.001	2.53 (1.61–3.96)	<0.001	1.67 (0.94–2.96)	0.08	4.75 (1.58–14.26)	0.01
cT+GGG	3.26 (2.64–4.02)	<0.001	3.33 (2.08–5.33)	<0.001	2.37 (1.32–4.26)	<0.001	3.4 (1.04–11.08)	0.04
cT+PSA+GGG	4.99 (3.85–6.47)	<0.001	3.14 (1.78–5.52)	<0.001	3.03 (1.51–6.09)	<0.001	7.22 (2.04–25.51)	<0.001

## Discussion

The D’Amico classification was introduced in 1998. Since its advent, the D’Amico classification has been universally adopted. However, multiple associations applied minor modifications that did not affect their performance at predicting CSM [12]. Although DHRCs have been analyzed in multiple studies, to date, the effect of R/E on the distribution of DHRCs as well as of their impact on CSM has not been tested. We hypothesized that R/E might affect the distribution of type and number of DHRCs. Moreover, we postulated that the effect of individual and/or combinations of two or three DHRCs may affect CSM differently according to R/E. We tested the above hypotheses within a large-scale, population-based cohort of RT-treated patients and made several noteworthy observations.

First, very important R/E differences with respect to the type and number of DHRCs exist according to R/E groups. Within the current study, the vast majority (75.8%) of HR patients harbored a single DHRC, while a smaller fraction (20.9%) harbored two DHRCs, and the smallest fraction (3.3%) exhibited all three DHRCs. Within patients with one DHRCs, Asian patients exhibited higher rates of GGG 4–5, low rates of PSA >20 ng/mL (14%), and lowest rates of T2c-T3 (10%). Conversely, African Americans exhibited lowest GGG 4–5 rates (43%), but highest rates of PSA >20 ng/mL rate (21%). These results might be of clinical importance. For example, a higher PSA at diagnosis might be a marker of delayed diagnosis in PCa, which in turn might be caused by known and described racial disparities in diagnosis and treatment of PCa [13–15]. Similarly, as our current study and previous ones reported biopsy GGG as the strongest predictor of CSM [16–22], knowing that HR Asian patients most frequently present with GGG 4–5 should guide treatment decision toward higher intensity approaches. Interestingly, combinations of two concomitant DHRCs exhibited substantially less pronounced differences than when individual DHRCs were examined. No differences were recorded for the concomitant presence of all three DHRCs across R/E. To the best of our knowledge, we are the first to describe the difference in the distribution of type and number of DHRCs among races/ethnicities.

Second, we tested the effect of type and number of DHRCs on absolute CSM rates, and we observed very important differences according to R/E. For example, Asians exhibited the lowest CSM rates of all R/Es, regardless of type and number of DHRCs. Moreover, Hispanic/Latinos showed the widest range of CSM rates (1.7–13.8%) and the highest CSM rate, when all three DHRCs were present. Interestingly, the magnitude of absolute CSM increase, according to the number of positive DHRCs, was very comparable within each R/E. Our observations are in line with previous analyses, which demonstrate a survival advantage in Asian patients with PCa compared to other R/E groups [6,9,23].

Subsequently, we analyzed the effect of type and number of DHRCs on relative CSM values, defined by hazard ratios, instead of absolute CSM values. Here, we observed an inverse phenomenon relative to our previous observations regarding absolute CSM rates. Specifically, hazard ratios were invariably highest in Asians than in the three other R/E groups, regardless of type and number of DHRCs. Highly interestingly, a clear rank order was observed within the three other R/E groups with respect to the magnitude of the effect of one, combinations of two, and all three DHRCs. Our observations are novel and of clinical importance, as, despite Asian patients displayed the lowest overall CSM, the stepwise effect according to the type and number of DHRCs is the highest. Therefore, it can be postulated that the modulation of treatment intensity according to type and number of DHRCs could be particularly important in Asian patients.

The PCa phenotype differences according to R/E observed in the current study need to be further examined within independent datasets to ensure validity. Unfortunately, not all large-scale population-based databases that provide adequate samples for R/E groups concomitantly include the required survival endpoints. For example, the National Cancer Database (NCDB) provides an even larger data pool than the SEER database, but lacks CSM vs. OCM stratification, which represents a critical pitfall [24]. However, the concept of R/E differences on the effect of established disease-specific risk factors on CSM has also been studied in other genitourinary cancers [6,23,25–30]. In consequence, the concept discussed in the current study is novel for PCa, but it is also generalizable, when other genitourinary cancers are considered.

Our study is not devoid of limitations. First, although white patients are well represented in the SEER database, the representation of African American, Hispanic/Latino, and Asian patients is suboptimal, with Asian patients representing the group with the smallest sample size. Therefore, oversampling of these patients should be encouraged in the future to allow better generalizability of observed findings within samples of African American, Hispanic/Latino, and Asian men. The second limitation is represented by the specific selection of RT patients. Third, absence of earlier cancer-control outcomes such as biochemical recurrence, progression-free survival, or metastatic progression may also be criticized. However, these endpoints are clearly not as definitive and not as established as the ultimate endpoint of CSM. Moreover, lack of central pathology for assessment of biopsy GGG represents a further limitation. However, the nature of the data reflects community practice patterns, where central pathology assessment of biopsy specimens is usually not applicable. Additionally, lack of information on the type and duration of androgen-deprivation and type and dosage of radiation therapy may also be limiting. Last but not least, the data are retrospective and are affected by biases that are operational in retrospective studies, which also apply to all other epidemiological analyses, such as those from SEER and NCDB.

To conclude, our study demonstrated important racial/ethnic differences in the distribution of type and number of DHRCs. Moreover and foremost, our study demonstrated lowest absolute CSM rates, but highest effect on relative CSM metrics in Asians. Conversely, the widest range of absolute CSM values was recorded in Hispanic/Latinos, but the lowest relative effect on relative CSM metrics. Finally, we recorded smallest differences in absolute and relative rates according to DHCRs between Caucasians and African Americans. These observations militate in favor of adopting R/E-specific interpretation of the effect of DHRCs on CSM in HR PCa patients treated with RT.

## Supplementary Material

Supplemental Material

## Approval of the research protocol by an Institutional Reviewer Board

All analyses and their reporting followed the Surveillance, Epidemiology, and End Results database (SEER) reporting guidelines. Due to the anonymously coded design of the SEER database, study-specific Institutional Review Board (IRB) ethics approval was not required.

## Abbreviations list:

CRR: competing risks regression

CSM: cancer-specific mortality

DHRCs: D’Amico high-risk criteria

GGG: Gleason Grade Group; HR: high-risk

IQRs: interquartile ranges

NCDB: National Cancer Database

OCM: other cause mortality

PCa: prostate cancer

RT: external beam radiotherapy
