# Supplementary figures and images for: Racial/ethnic disparities in the distribution and effect of type and number of high-risk criteria on mortality in prostate cancer patients treated with radiotherapy
Source: Arab J Urol. 2022 Nov 21;21(3):135–41. doi: 10.1080/2090598X.2022.2148867 (PMC10373609; doi:10.1080/2090598X.2022.2148867)

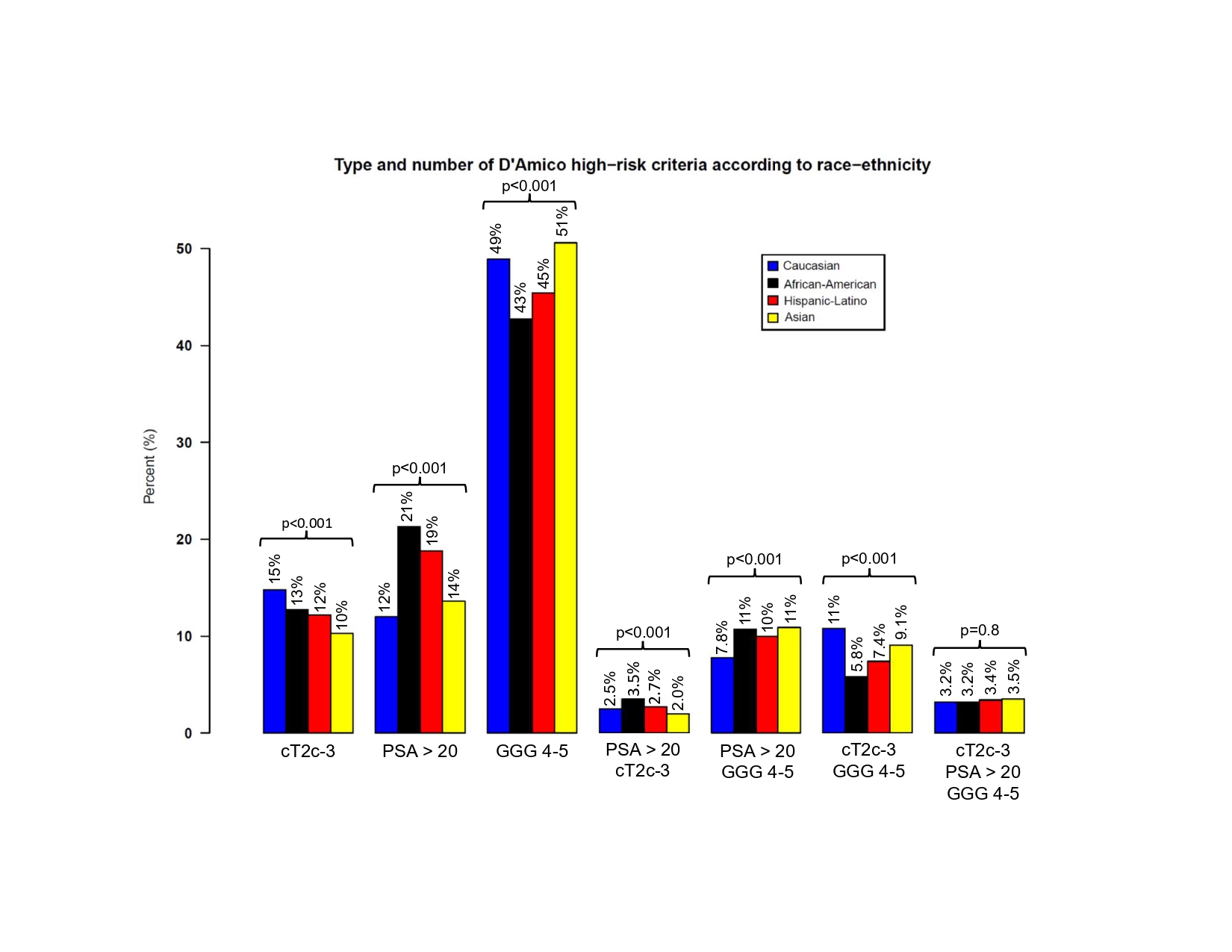

Supplement: Supplemental Material [file TAJU_A_2148867_SM2789.jpg]
